# Molecular Diversity and Agronomic Performance of Sesame (*Sesamum indicum*) Cultivars in Benin: Local Cultivars and Lines Introduced From China

**DOI:** 10.1002/pei3.70024

**Published:** 2024-12-24

**Authors:** Christel Ferréol Azon, Nicodème V. Fassinou Hotegni, Charlotte O. Adjé, Lewis Spencer Gnanglè, Evelyn Benjamin, Ruvarashe Loveness Mhuruyengwe, Abdou Mouizz Salaou, Aristide Carlos Houdegbe, Deedi Olga Sogbohossou, Paulin Sedah, Komivi Dossa, Clément Agbangla, Florent J. B. Quenum, Enoch G. Achigan‐Dako

**Affiliations:** ^1^ Genetics, Biotechnology and Seed Science Unit (GBioS), Laboratory of Crop Production, Physiology and Plant Breeding, Faculty of Agricultural Sciences University of Abomey‐Calavi Cotonou Republic of Benin; ^2^ Department of Biotechnology, Faculty of Science Ebonyi State University Abakaliki Nigeria; ^3^ Department of Crop Science University of Zimbabwe Harare Zimbabwe; ^4^ CIRAD, UMR AGAP Institut Montpellier France; ^5^ UMR AGAP Institut, Université Montpellier, CIRAD, INRAE, Institut Agro Montpellier France; ^6^ Laboratory of Molecular Biology and Genome Analysis, Faculty of Sciences and Techniques University of Abomey‐Calavi Cotonou Republic of Benin

**Keywords:** agromorphological variation, genetic diversity and differentiation, molecular variation, sesame, simple sequence repeat (SSR)

## Abstract

Sesame cultivation was until recently restricted to the northwestern part of Benin. The yield is relatively low, as there are no improved varieties introduced and widely adopted so far. This study aimed to assess the molecular diversity, genetic differentiation, and the agronomic performance of a collection of local cultivars and introduced lines of sesame from China. The agronomic evaluation was conducted across eight environments during the 2020 cropping season using 14 descriptors on 19 accessions, including 6 introduced lines arranged in a randomized complete bloc design. Twelve simple sequence repeat markers were used to assess the molecular diversity. The analysis of variance showed significant variation among accessions for all the traits, except the number of lodges per capsule. Principal component analysis (PCA) followed by hierarchical clustering indicated that the accessions could be classified into three groups. The first group included accessions from China with the local accession SI09, characterized by early flowering and low seed yields (on average 380.13 kg ha^−1^). The second group included late flowering accessions and intermediate seed yield (on average 548.68 kg ha^−1^). The third group included higher yielding accessions (on average 715.7 kg ha^−1^). The PCA identified key traits such as days to 50% emergence, days to 50% flowering, collar diameter, plant height, number of branches, and seed yield as the most discriminative among accessions for agromorphological characterization. The SSR markers were polymorphic, with polymorphic information content values between 0.17 and 0.92. A total of 62 alleles were detected, with each locus exhibiting 2 to 15 alleles. The gene diversity ranged from 0.18 to 0.92, with an average value of 0.55. Cluster analysis based on the unweighted pair group method with arithmetic mean revealed that accessions were grouped in three clusters, with the coefficients of similarity/dissimilarity ranging between 0.60 and 0.92. Most of the Chinese lines were clustered together, except accession Y01. This study provided useful knowledge about local sesame cultivars in Benin and their similarities and differences with the lines introduced from China, therefore contributing to the advancement of the sesame‐breeding program in the country.

## 1 Introduction

Sesame ( *Sesamum indicum* L.) is an annual diploid plant species belonging to the Pedaliaceae family, with a chromosome number of 2 *n* = 26. It is ranked ninth among the top 13 oilseed crops, as widely cultivated as an oil seed crop and contributes to 90% of global edible oil production (Degefa 2019). Sesame seeds are known for their high oil content, between 50% and 60%, positioning them as “queen of oilseed crops” (Gavrilova et al. 2020; Iqbal et al. 2018). Sesame oil is rich in proteins, carbohydrates, minerals, and polyunsaturated fatty acids and is a unique source of antioxidant called Sesamin and sesamolin (Biswas et al. 2018). This significance has led to a surge in both its consumption and production, as market research indicates a growing trend in global sesame demand (Sirany and Tadele 2022). Projections indicate a potential supply gap of 3 million tonnes by 2040, indicating a critical need to increase sesame production to meet anticipated future demands (Rahman et al. 2020).

The primary regions for sesame cultivation are Asia and Africa, which together represent over 90% of the world‐cultivated areas. Sesame is cultivated worldwide in various climatic conditions such as tropical and semi‐tropical areas, making it adaptable to different environments (Stavridou et al. 2021). According to Misganaw, Mekbib, and Wakjira (2015), sesame thrives relatively well under high temperatures and can be grown using residual soil moisture. Depending on the country, farmers may choose to grow either traditional landraces or improved varieties. Countries with extensive experience in sesame cultivation, such as China, India, and Japan in Asia, as well as Senegal, Burkina Faso, Mali, and Ethiopia in Africa, have developed improved varieties (Boru 2021; Teklu, Shimelis, and Abady 2022). However, in Burkina Faso, Nigeria, Niger, and Senegal, despite the improved varieties developed, landraces continue to be utilized (Gildemacher et al. 2015; Sene et al. 2018; Teklu, Shimelis, and Abady 2022).

The morphology of the sesame crop, regardless of the varieties and landraces, is assessed using morphological descriptors such as the plant height (PH), number of branches (NB), stem color, types of leaves, number of capsules per plant (NCP), and seed color. Sesame PH varies between 0.5 and 2.5 m and can be branched or non‐branched depending on the genotypes (Zewdie and Tehulie 2019). Agronomic parameters, such as the number of capsules and the number of seeds per capsule (NSC), vary across accessions, and constitute, in many cases, discriminatory characters among accessions (Langham 2020). According to Myint et al. (2020), sesame breeding should consider agronomic traits primarily for increased yield, increased oil content, and yield‐related traits. The same study indicated that China had the highest average sesame yield (1223 kg ha^−1^), followed by Nigeria (729 kg ha^−1^) and Tanzania (720 kg ha^−1^).

Compared with other economically significant crops, sesame is still a poorly investigated crop, particularly in various African countries, highlighting the need for increased research efforts (Harfi et al. 2021). Research related to classical breeding and genetic era in sesame started 2000, and focusing on interspecific crossing, development of molecular markers, genetic diversity study, low‐resolution genetic maps etc. Dossa et al. (2017). Understanding the genetic diversity in the species is key for selecting the best genotypes for breeding programs and is essential for achieving and sustaining high productivity (Kabi et al. 2020). Developing a breeding program for most crops requires gathering germplasm through either traditional landraces and related wild species or improved varieties, all of which are important sources of genetic diversity for breeders and form the backbone of agricultural production (Abate and Mekbib 2015).

Genetic diversity assessment and conservation is particularly urgent nowadays due to the widespread loss of species globally. Previous studies have shown that sesame landraces have an extensive phenotypic and genotypic diversity (Stavridou et al. 2021). Genetic diversity studies, including agromorphological and molecular levels, were carried out worldwide in sesame to identify superior germplasm. According to Pandey et al. (2015), the assessment of existing genetic variation by combining both agromorphological and molecular characteristics in indigenous and exotic collections of sesame is essential for developing effective management strategies for crop improvement. Many molecular markers were used in sesame genetic diversity assessments such as Random Amplified DNA Polymorphism (RAPD), Amplified Fragment Length Polymorphism (AFLP), genome Simple Sequence Repeat (gSSR), Simple Sequence Repeat (SSR), Single Nucleotide Polymorphism (SNP) etc,. These markers have successfully revealed the diversity present among sesame accessions (Adu‐Gyamfi, Prempeh, and Zakaria 2019; Stavridou et al. 2021). In most of the cases, SSR markers were favored due to their facility of reproduction, hypervariability, effectiveness, and codominance inheritance (Wang et al. 2023; Zhang et al. 2010).

In Benin, sesame is primarily grown as a diversification crop, yet it has received little attention from research. Given the increasing importance of the crop, there is a need to develop cultivars that are adapted to the specific conditions of the country. As suggested by Adu‐Gyamfi, Prempeh, and Zakaria (2019), the development of new varieties requires the collection and characterization of genotypes to determine the level of diversity present. Understanding this diversity is critical for the improvement of desirable traits in the crop.

The present study has addressed the following research questions: Is there any variation among accessions for the studied traits across environments? Are there any significant differences in the results of agromorphological versus molecular analyses among the accessions? How do local accessions perform compared with introduced lines of sesame? Is the collection used effective for selecting the best parental lines for the initiation of a breeding program?

The study aimed at assessing the genetic diversity and differentiation within a core collection of Benin and Chinese sesame accessions for selection of the most promising accessions for inclusion in a breeding program. The specific objectives were to (1) assess agromorphological diversity among sesame accessions across eight distinct environments in Benin, (2) analyze molecular diversity among accessions using SSR markers, (3) investigate the similarities between Benin and Chinese accessions, and (4) select the top‐performing accessions for breeding purposes. We hypothesized that (1) there is variability among accessions regarding agromorphological traits, (2) SSR markers indicate high genetic diversity among accessions, (3) there are significant differences between Benin and Chinese accessions in terms of agromorphological and molecular diversity, and (4) the accessions used in this study perform well and can be selected for breeding program.

## 2 Materials and Methods

### 2.1 Agromorphological Characterization

#### 2.1.1 Plant Material and Experimental Sites

The plant material used in this study consists of 19 cultivars, including 13 accessions collected from different sesame production areas in Benin and six lines introduced from China (Table 1).

**TABLE 1:** Source, origin, types, and seed color of 19 sesame cultivars used in this study.

No	Accessions	Status/Pedigree	Origin	Seed color	Locality of origin
1	SI01	Farmers seed	Benin	White	Boukoumbe
2	SI02	Farmers seed	Benin	White	Bembereke
3	SI03	Farmers seed	Benin	Red	Tanguieta
4	SI04	Farmers seed	Benin	White	Tanguieta
5	SI05	Farmers seed	Benin	White	Banikoara
6	SI06	Farmers seed	Benin	White	Tanguieta
7	SI07	Farmers seed	Benin	Red	Boukoumbe
8	SI08	Farmers seed	Benin	Red	Boukoumbe
9	SI09	Farmers seed	Benin	Black	Tanguieta
10	SI10	Farmers seed	Benin	White	Karimama
11	SI11	Farmers seed	Benin	White	Karimama
12	SI12	Farmers seed	Benin	White	Kerou
13	SI13	Farmers seed	Benin	White	Pehunco
14	Y01	Advanced line	China	White	Yanzhuang
15	Y02	Advanced line	China	White	Yanzhuang
16	Y03	Advanced line	China	White	Yanzhuang
17	Y04	Advanced line	China	White	Yanzhuang
18	Y05	Advanced line	China	White	Yanzhuang
19	Y06	Advanced line	China	White	Yanzhuang

#### 2.1.2 Experimental Sites and Climatic Conditions

**FIGURE 1:**
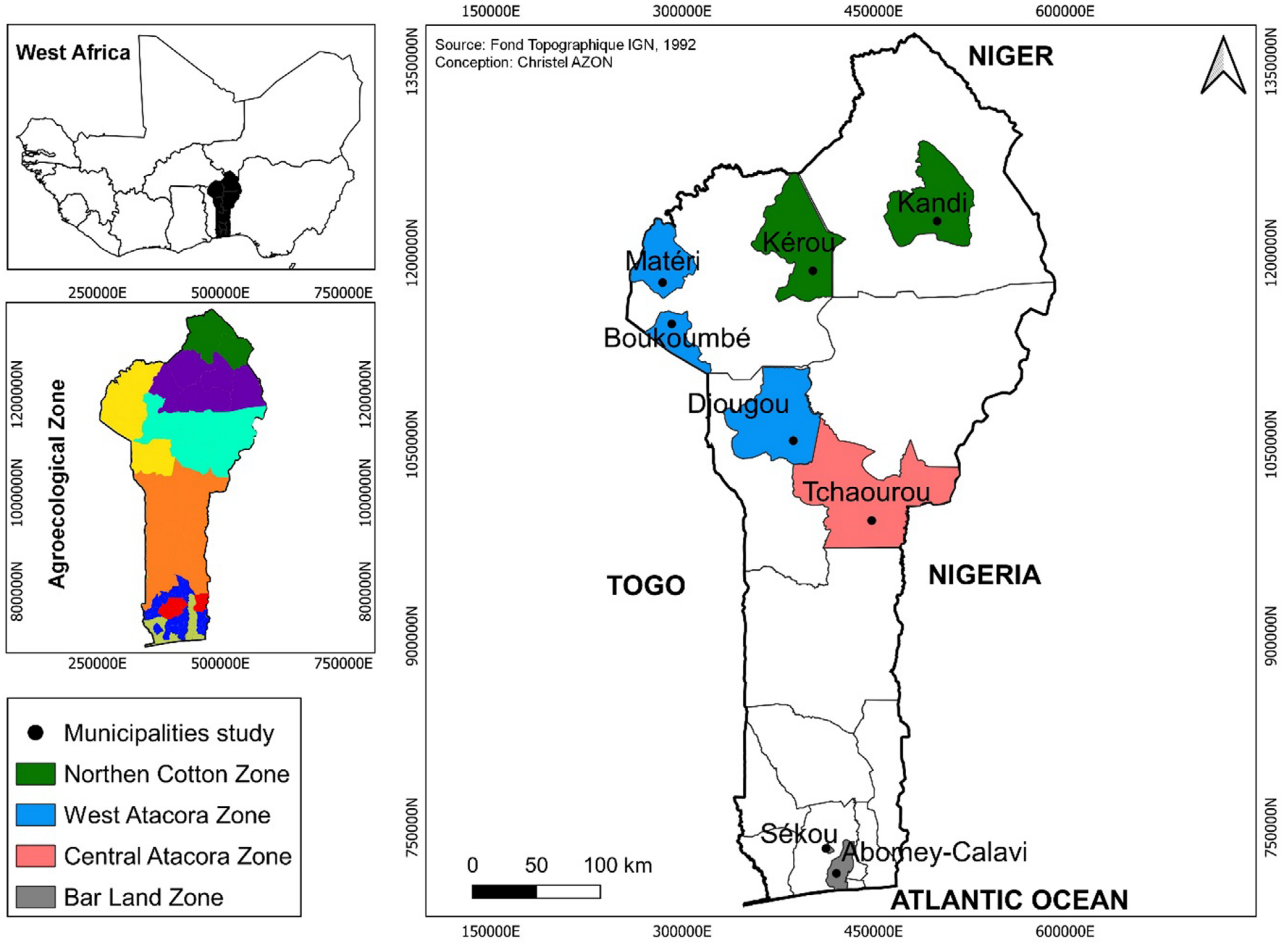
Distribution of study environments across Benin's agroecological zones.

The evaluation of the agromorphological diversity was conducted using a randomized complete block design with four replications across eight environments in the Republic of Benin. Each plot was composed of 26 plants arranged in two rows, with 13 plants in each row. The spacing between plants within a row was of 0.30 m, and the distance between rows was 0.5 m. The experimental sites were Abomey‐Calavi, Sékou, Tchaourou, Djougou, Boukoumbé, Matéri, Kérou, and Kandi (Figure 1).

The climatic conditions recorded during the trials and characteristics of the eight environments are presented in Table 2.

**TABLE 2:** Experimental sites characteristics and climatic conditions during the experimentation period.

Sites	Latitude (N°)	Longitude (E°)	Altitude (m)	Rainfall (mm)	Min–max temperature (°C)	Relative humidity	Soil types
Abomey‐Calavi	6°25′	2°20′	17	606.64	24.25–29.38	76.5	Fertile, alluvial soils
Sékou	6°37′	2°13′	90	266.96	23.22–31.30	73.11	Predominantly ferralitic (red soil) (world reference base for soil resources)
Tchaourou	9°17′	2°24′	394	455.6	20.4–32.2	72.66	Ferruginous on crystalline base
Djougou	9°41′	1°40′	444	469	19.5–31.9	61	Ferruginous often on deep base
Boukoumbé	10°16′	0°56′	200	425	19.3–31.7	63	Sandy clay
Matéri	11°03′	0°58′	184	325	20.8–33.5	62.5	Ferruginous often on deep base
Kérou	10°49′	2°6′	310	348.61	19.64–33.65	61.9	Tropical ferruginous on a crystalline base with proportions of deep and little concreted soils
Kandi	11°08′	2°56′	294	319.2	19.84–33.85	67.66	Ferruginous on crystalline base

*Note:* Information about characteristics of agroecological zones was collected from Nacoulma and Guigma (2015) and Daasi et al. (2021). Climate data were collected from Trans‐African Hydro‐Meteorological Observatory (TAHMO) in 2020.°C (Degree Celsius), mm (millimeter), m (meter), N° degree North, E° degree East.

The selected sites were distributed across the southern, central, and northern parts of the country. The southern sites, specifically Abomey‐Calavi and Sékou, exhibited high relative humidity levels. Abomey‐Calavi recorded the highest rainfall at 606.64 mm, whereas Sékou experienced the lowest rainfall (266.96 mm) despite its high relative humidity level. In the central and northern regions, the average rainfall varied from 469 to 319.2 mm. Additionally, the soil was predominantly ferruginous in the northern region, including Djougou, Boukoumbé, Matéri, Kérou, and Kandi.

#### 2.1.3 Agromorphological Data Collection

Quantitative traits related to flowering, growth, and yield components were collected on the 10 central plants. The parameters recorded included days to 50% emergence (DE), days to first flowering (DFF), days to 50% flowering (D50F), PH, NB, collar diameter (CD), NCP, capsule length (CL), capsule width (CW), NSC, number of lodges per capsule (NL), thousand‐seed weight (TSW), and seed yield (SY) (Table S3).

### 2.2 Molecular Characterization

Young leaves were collected 2 weeks after sowing and ground for DNA extraction using the CTAB (cetyltrimethylammonium bromide) protocol (Dossa et al. 2016; Zangui et al. 2020). DNA quality was checked and quantified using Nanodrop Lite 2000 Spectrophotometer (Thermo Scientific). PCR (polymerase chain reaction) was carried out in a reaction volume of 20 μL containing 2,5 μL of 10× PCR buffer concentration (B90145), 1.25 μL of 25 mM of (magnesium dichloride) MgCl_2_, 0.75 μL of 0.1% bovine serum albumin (BSA), 0.75 μL of 200 μM dNTP mix, 2.5 μL of 0.2 μM each of forward and reverse primer, 0.2 U Taq Polymerase (OneTaq Inqaba), 3 μL of 25 ng/μL template DNA, and completed molecular water. The amplification was carried out using the PCR program according to Dossa et al. (2016): 94°C for 4 min (stage 1); then 35 cycles each of 94°C for 30 s, 55°C for 30 s, and 72°C for 30 s (stage 2); and a final extension of 72°C for 10 min and cooling (stage 3). The characteristics of the 12 microsatellite SSR primers used in our study are presented in Table 3. The alleles scoring was performed manually based on the presence of a sized allele in each accession. The presence of a band was denoted as 1 and the absence as 0.

### 2.3 Statistical Analysis

The data were analyzed using descriptive statistics, including the calculation of means and the coefficient of variation. A normality test was performed for all the studied traits. We used the analysis of variance (ANOVA) for normally distributed variables and Kruskal–Wallis's test for nonnormal variables to test if there is any difference among accessions within and across locations. The Tukey's test was then performed to compare the mean of accessions for the traits across environments. The Spearman correlation test was carried out to assess the relationships among studied traits. A principal component analysis (PCA) was performed with all the recorded traits and a dendrogram was generated to classify accessions in different groups.

For molecular analysis, we computed genetic diversity indices, including number of different alleles (Na), number of effective alleles (Ne), expected heterozygosity (He), and Shannon Index (I), using the GenAlex software. Major allele frequency (MAF), number of alleles (NA) per locus, polymorphic information content (PIC), and gene diversity (GD) were carried out using PowerMarker software version 3.25. To further elucidate the clustering patterns, analysis of molecular variance (AMOVA) between populations and principal coordinate analysis (PCoA) were performed with GenAlex software version 6.51. Accessions were clustered following the UPGMA method in NTSYS software version 2.10. The Chinese line Y01 has been used as a check for quality assurance. The similarity between agromorphological and molecular data was estimated using the Mantel test with the package “ade4” as performed by Malek et al. (2021).

## 3 Results

### 3.1 Agromorphological Characterization

#### 3.1.1 Descriptive Statistics and ANOVA

**FIGURE 2:**
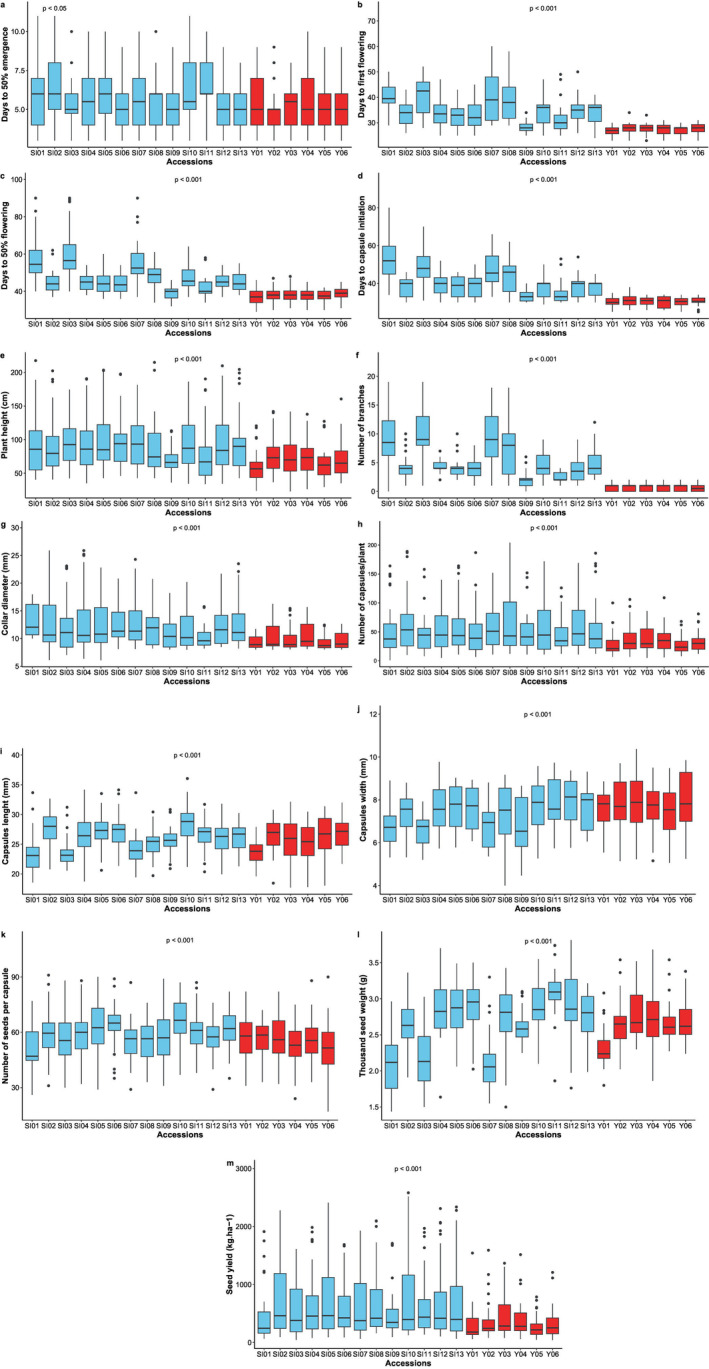
Box plots showing natural variation in studied traits among local and Chinese accessions of *Sesamum indicum* evaluated in eight locations in Benin. a‐ variation among local and Chinese accessions for days to 50% germination, b‐ variation among local and Chinese accessions for days to first flowering, c‐ variation among local and Chinese accessions for days to 50% flowering, d‐ variation among local and Chinese accessions for days to capsule initiation, e‐ variation among local and Chinese accessions for plant height, f‐ variation among local and Chinese accessions for number of branches, g‐ variation among local and Chinese accessions for collar diameter, h‐ variation among local and Chinese accessions for number of capsules per plant, i‐ variation among local and Chinese accessions for capsule lenght, j‐ variation among local and Chinese accessions for capsule width, k‐ variation among local and Chinese accessions for number of seeds per plant, l‐ variation among local and Chinese accessions for thousand seed weight, m‐ variation among local and Chinese accessions for seed yield. *p* < 0.05 significant difference, *p* < 0.001 highly significant difference, cm (centimeter), g (gram), kg ha^−1^ (kilogram per hectare), mm (millimeter).

The variation in traits per environment showed that there is significant difference among accessions for most studied traits. However, the NL did not differ, regardless of the environment. In addition, the number of days for 50% emergence and the NCP did not significantly differ in Tchaourou and Materi locations (Table S4). The ANOVA performed across environments showed that there was a significant difference (*p* < 0.001) among accessions for all quantitative traits studied except the NL. D50F occurred earlier in the Chinese accessions than in the local accessions, whereas late flowering accessions were observed in the local accessions (SI01, SI03, and SI07) (Figure 2c). The mean value for D50F across all accessions ranged from 36.4 to 61 DAS (days after sowing; Table S5).

The accessions that were introduced exhibited lower PH measurements compared to the local accessions, which recorded higher values (Figure 2e). The local accessions also demonstrated the greatest NB, with SI03 achieving the highest count, whereas the Chinese accessions (Y01, Y02, Y03, Y04, Y05, and Y06) had the lowest branch numbers (Figure 2f). The maximum number of capsules per plant was obtained among the local accessions, with the highest values recorded with SI08, SI02, and SI12 (Figure 2h). SI08 showed the highest average NCP (66.60), followed by SI02 (65.8) and SI12 (64.44). In contrast, the lowest average values were recorded with Y01 (27.4) and Y05 (27.19) (Table S5). The highest NSC was observed with local accessions with the lowest values recorded in the Chinese accessions (Figure 2k). Considering the TSW, local accessions showed both the lowest and highest values, with SI01 and SI11, respectively, representing these extremes (Figure 2l). SI11 recorded a high value (3.1), whereas SI01 and SI07 had low values of 2.11 and 2.12, respectively. The local accessions showed the highest values of SY, whereas the lowest values were obtained for Chinese accessions (Figure 2m).

#### 3.1.2 PCA and Clustering of Accessions Using Agromorphological Traits

The PCA for agromorphological traits showed that the first two components explained 75.52% of the total variation (Table S1). The first component is positively correlated with several traits, such as DE, D50F, CD, PH, NB, and SY, per hectare. The second component is positively correlated with DE, PH, CD, NCP, CL, CW, NSC, TSW, and SY (Table S2).

**FIGURE 3:**
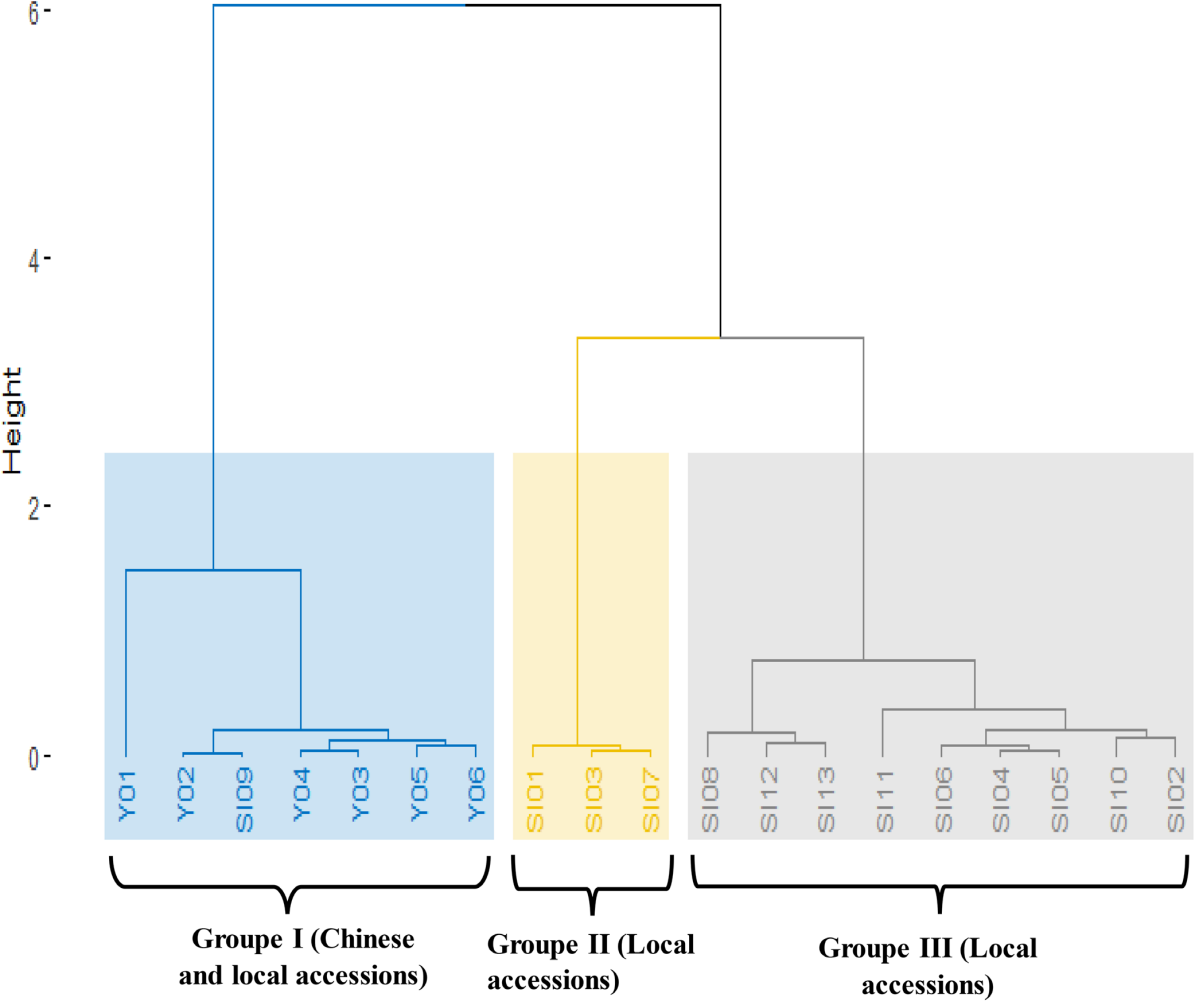
Dendrogram based on Euclidian distance through the Neighbor‐Joining method of quantitative characters in 19 sesame accessions evaluated across eight locations in Benin.

The accessions were classified into three groups (Figure 3). The first group included seven accessions, namely, the accessions from China and one accession from Benin (SI09). The second group included three accessions, namely, SI01, SI03, and SI07. The last group, which had a large number of accessions, included nine local accessions. The first group is characterized by early flowering accessions. The average NCP for this group ranged from 27.2 to 37.2. The group included accessions with low SY with an average value of 380.13 kg ha^−1^. The second group comprises three accessions (SI01, SI03, and SI07), which are late flowering (average duration of 58.90 DAS). The NB was on average 9.34. The average NSC in this group is 53.44, whereas the average value for SY is 548.68 kg ha^−1^. The third group is characterized by accessions with an average value of 94.04 cm and 45 DAS respectively for PH and D50F. The NSC is 69.6 and the average SY is 715.7 kg ha−1. This group had the highest value for SY.

#### 3.1.3 Relationship Between the Studied Traits

**FIGURE 4:**
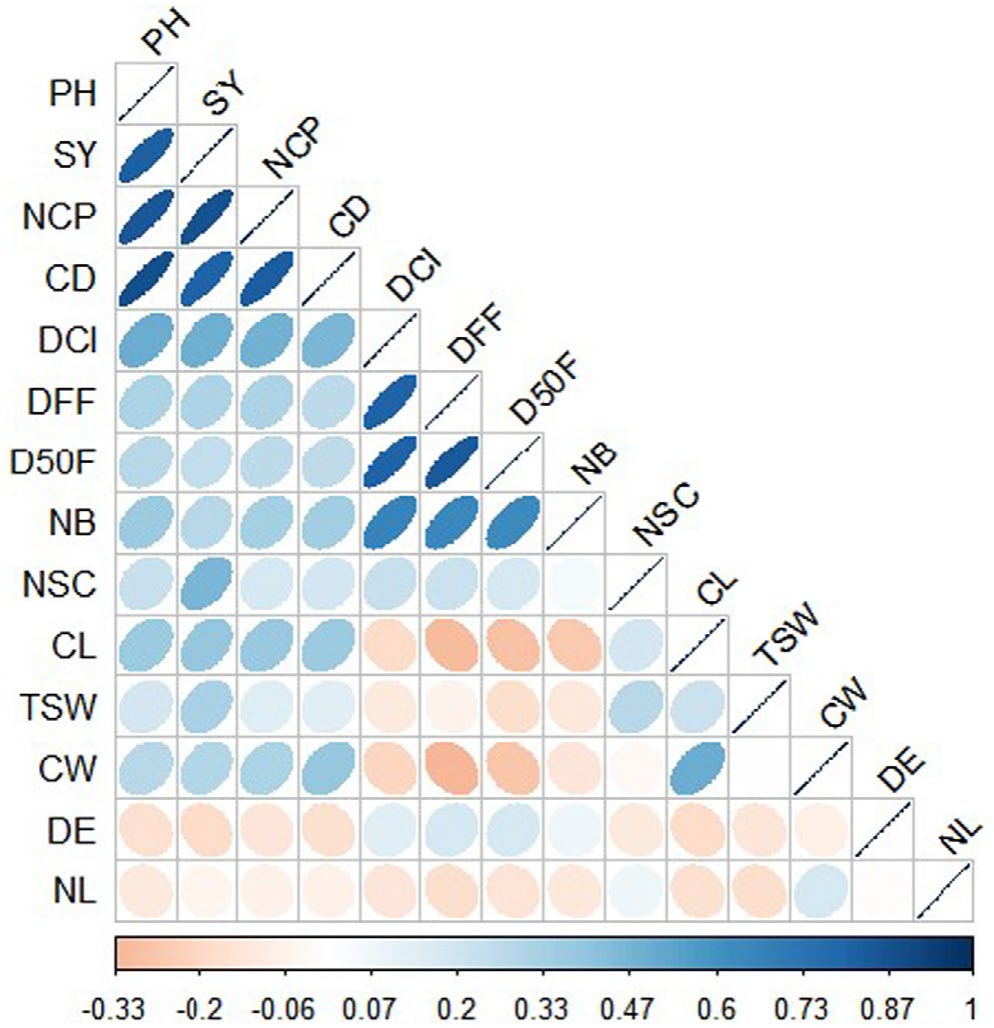
Spearman correlation matrix among 14 quantitative traits of sesame cultivars. DE, days to 50% emergence; DFF, days to first flowering; D50F, days to 50% flowering; PH, plant height; NB, number of branches; CD, collar diameter; NC, number of capsules; CL, capsule length; CW, capsule width; NSC, number of seeds per capsule; NL, number of lodges; TSW, thousand‐seed weight; SY, seed yield. Colors show the level of correlations (r) from positive (blue) to negative (red).

The correlation matrix between studied traits is presented in Figure 4. PH was highly and positively correlated with SY (*r* = 0.86***) and NCP (*r* = 0.81***). A positive and significant correlation was observed between PH and NSC (*r* = 0.28*) and PH and TSW (*r* = 0.23*). SY was positively and highly correlated with the NCP (*r* = 0.86***). There was also a positive but significant association between SY and the NSC (*r* = 0.43*) and TSW (*r* = 0.33*). A positive and high significant correlation was found between D50F and NB (*r* = 0.63***) and between D50F and DFF (*r* = 0.83***). A negative and significant correlation was found between NB and the TSW (*r* = −0.05**). Also, the correlation between the NB and CL and the NB and CW is negative and significant (*r* = −0.25** and *r* = −0.14**, respectively, for CL and CW).

### 3.2 Molecular Analysis

#### 3.2.1 Assessment of Genetic Parameters

**FIGURE 5:**
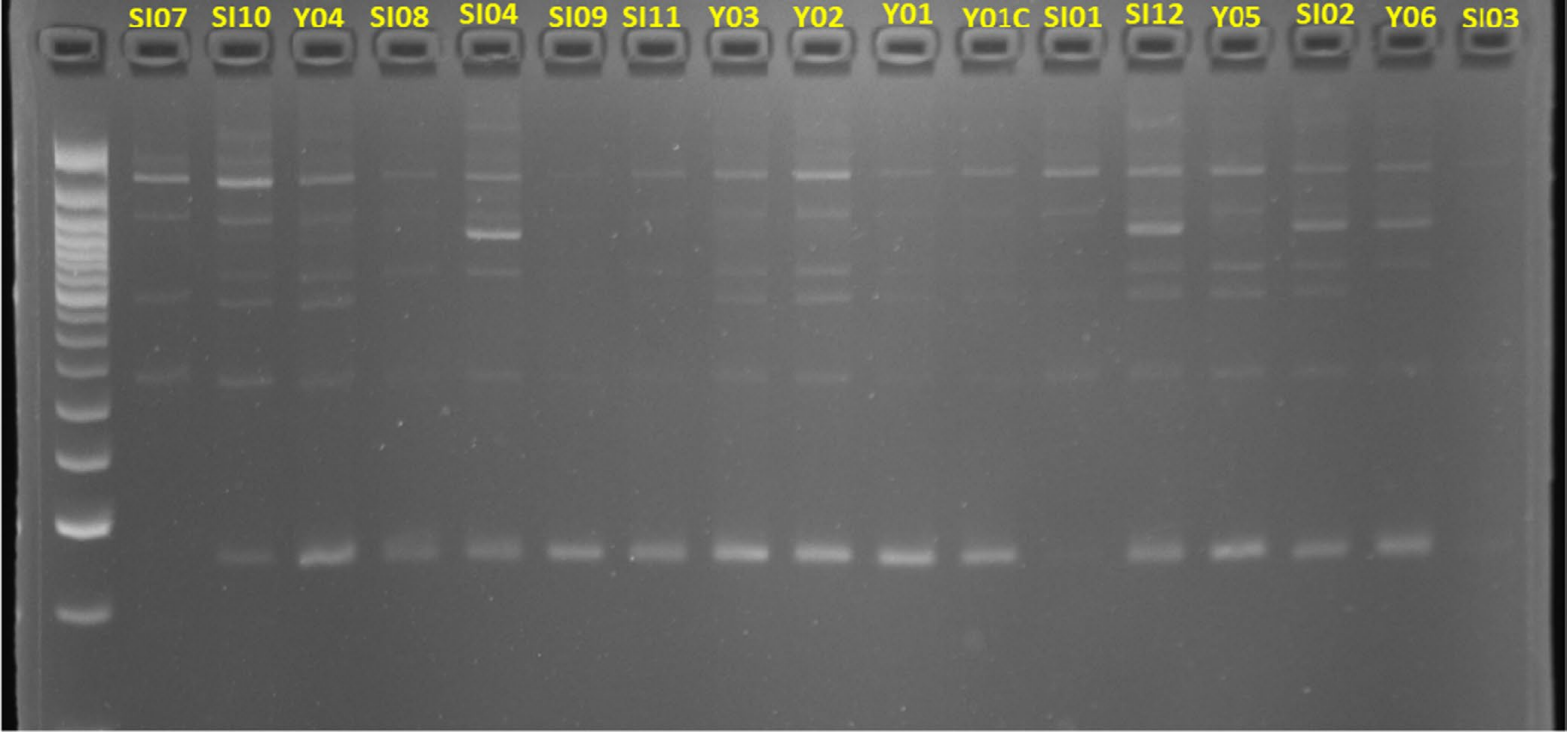
SSR genomic profile of the 17 sesame accessions (including Y01C as check) out of the 19 generated by the marker 3 (ZMM 4604).

Twelve SSR markers were used to assess the genetic diversity of our 19 sesame accessions. Table 3 shows the value of genetic parameters, namely, MAF, NA, GD, and PIC. All the 12 markers used in this study were polymorphic. The NA was 62 and ranged from 2 to 15 alleles per locus with a mean of 5.16. The MAF ranged from 0.1 to 0.89, with an average of 0.50. The GD varied from 0.18 (M12) to 0.92 (M9) with an average of 059. The average value obtained for PIC was 0.56 with a minimum value of 0.17 obtained for M12 and a maximum of 0.92 for M9. Figure 5 shows an example of a molecular profile revealed by the marker M3 (ZMM4604).

**TABLE 3:** Description of 12 SSR markers used to assess the genetic diversity in 19 sesame accessions from Benin and China.

N°	Primer name	Code	Location (cM)	Forward sequence	Reverse sequence	Size (bp)	Major allele frequency	Number of alleles	Gene diversity	Polymorphic information content
1	ZMM3986	M1	88.531	CCAATCCACAACATATGCCA	AAAGGTTGGGGTGAACAGTG	213	0.63	6	0.56	0.53
2	ZMM4145	M2	0	AGGAGGGTGGCTAAATT GCT	ACTTGACCATGTTTGGAG CC	219	0.26	8	0.84	0.83
3	ZMM4604	M3	21.058	ATCCCTTGGGTAGAGGGAGA	TTTGGGCATTTTGGAAACTC	177	0.1	15	0.92	0.92
4	ZMM4634	M4	39.704	TTTTCCTCCTGTTTCTTG GG	TCTCTGTGTGTACATGTAG CTTGTG	256	0.73	3	0.42	0.38
5	ZMM4657	M5	42.779	CACAGCTGTTGCAGAAA ACA	TTTGGCACATGCTTGTGA AT	264	0.31	6	0.77	0.73
6	ZMM4660	M6	37.688	TGCAAAACCGTAACACT CCA	CGAGCTCTACGTGTTTAG TGAAAA	265	0.31	8	0.79	0.77
7	ZMM4682	M7	56.398	ATGTGCCAATAACCACC GTT	AACAAGTCAATTGGGGTC AAA	274	0.52	2	0.49	0.37
8	ZMM4689	M8	52.234	CCACCTTTTAACCCCAA TCA	TGAGGGTTTGAATCCGTC TC	159	0.31	4	0.73	0.68
9	ZMM4702	M9	118.082	CAGATTAAAATTGCCAC CGC	TACAAGCCAGGTTTTGAG GG	122	0.73	2	0.38	0.31
10	ZMM4803	M10	141.156	TGCATGAGCTAAGGGA AAGG	TGGTGGCAATTTGCAAGT AA	268	0.36	3	0.66	0.59
11	ZMM4814	M11	55.905	CATCAAACTTTAGGAGA GGAAAGTG	TGGTGTATGCGGGATATT TG	250	0.78	3	0.35	0.32
12	ZMM4893	M12	39.414	GACTTTAGCGGGAGCAC AAC	GGAGTAGGCCTTTCATTC TTCA	198	0.89	2	0.18	0.17
Means							0.5	5.16	0.59	0.55

Abbreviations: bp, pair base; cM, centimorgan; SSR, simple sequence repeat.

The genetic diversity indices were calculated to assess the allelic patterns across the two populations. The mean values for the number of samples (N), number of different alleles (Na), number of effective alleles (Ne), Shannon's index (I), expected heterozygosity (He), and unbiased heterozygosity (uHe) between population and across population are presented in Table 4. The genetic diversity indices in the Benin population (Na = 1.83, Ne = 1.49) are higher than those in the Chinese population (Na = 1.47, Ne = 1.27). Also, the other indices, such as Shannon's index (I), expected heterozygosity, and unbiased expected heterozygosity, are higher in the population from Benin (I = 0.45, He = 0.30, uHe = 0.31) than the population from China (I = 0.26, He = 0.16, uHe = 0.18).

**TABLE 4:** Genetic diversity indices among 19 local cultivars and introduced lines of sesame in Benin.

Pop	N	Na	Ne	I	He	uHe
Benin	13	1.83	1.49	0.45	0.30	0.31
China	6	1.47	1.27	0.26	0.16	0.18
Mean	9.5	1.62	1.37	0.36	0.27	0.24

Abbreviations: He, expected heterozygosity; I, Shannon's index; N, number of samples; Na, number of different alleles; Ne, number of effective alleles; Pop, population; uHe, unbiased expected heterozygosity.

#### 3.2.2 Analysis of Molecular Variance

The variation among and within populations was done through the AMOVA (Table 5). The total variance showed that 92% of the variation was obtained within populations, whereas only 8% was obtained among populations.

**TABLE 5:** Summary of the analysis of molecular variance (AMOVA).

Source	df	SS	MS	Est. Var.	%
Among populations	1	6.564	6.564	0.240	8%
Within populations	36	94.436	2.623	2.623	92%
Total	37	101.000		2.863	100%

Abbreviations: Est. Var., estimated variance; df, degree of freedom; MS, mean squared deviation; SS, sum of squared deviation.

#### 3.2.3 PCoA and Clustering of Sesame Accessions

**FIGURE 6:**
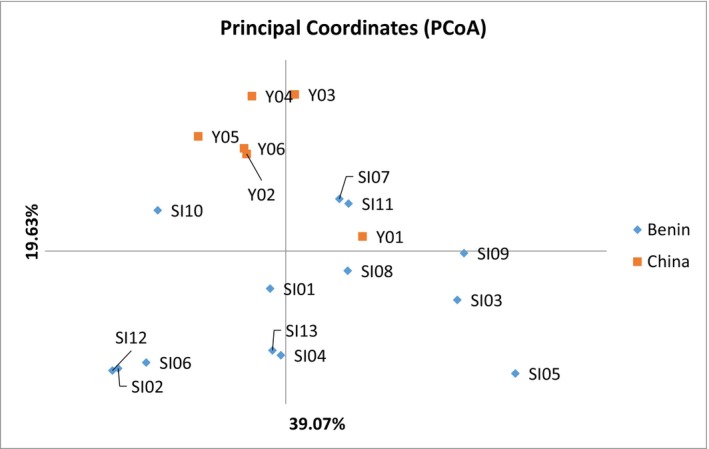
Principal coordinates analysis of the 19 sesame accessions based on SSR markers.

The genetic differentiation among accessions was carried out through PCoA and presented in Figure 6. The PCoA with SSR markers highlights the diversity among accessions. The first two axes explain 58.70% of the total variation. The first quadrant included accessions Y02, Y04, Y05, Y06, and SI10. The second quadrant included accessions SI07, SI11, Y01. In the third quadrant, accessions found were SI09, SI03, SI05, and SI08, whereas in the fourth quadrant, accessions were SI12, SI06, SI02, SI13, and SI04.

**FIGURE 7:**
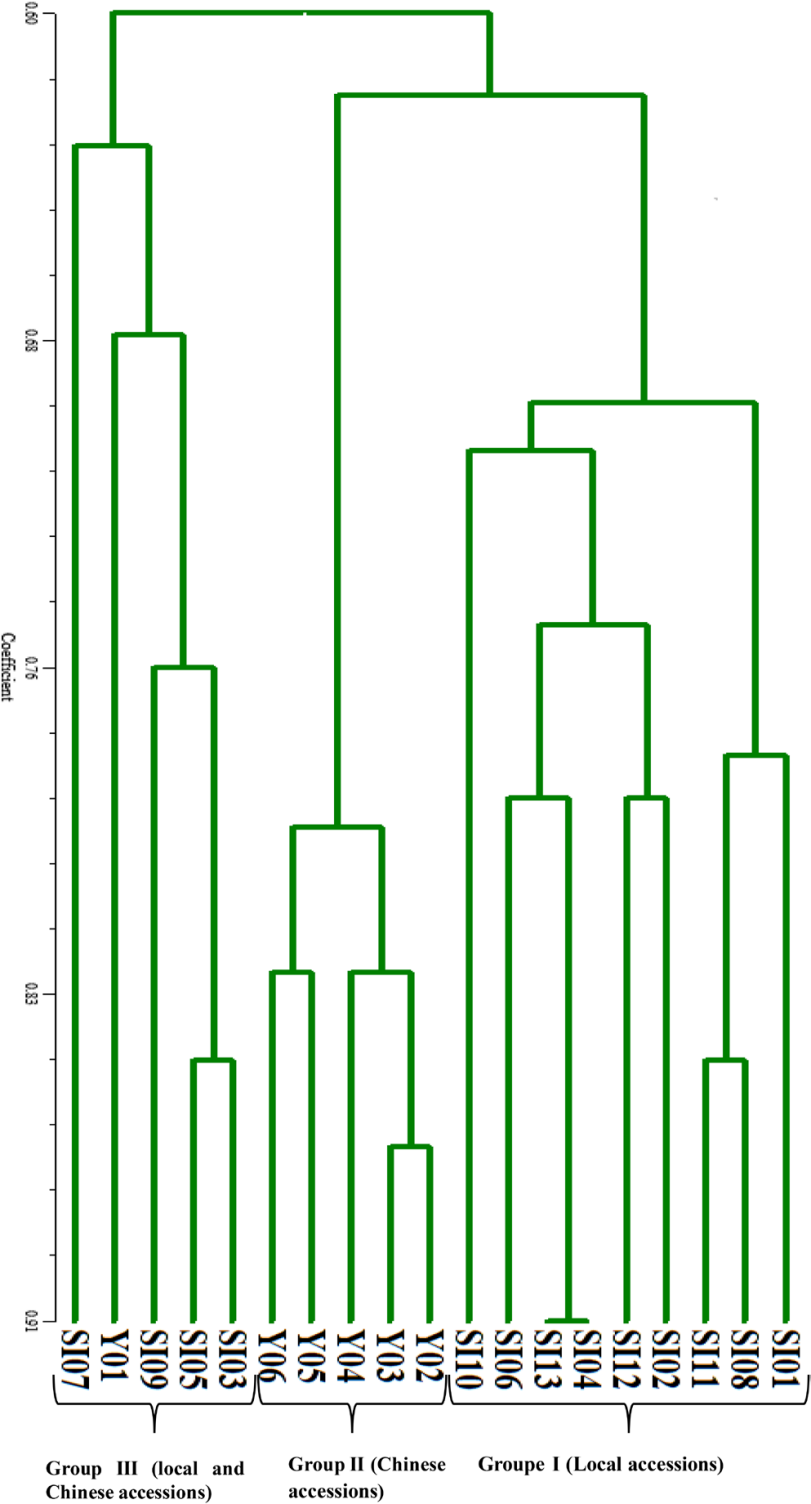
Dendrogram derived from the cluster analysis of 19 sesame accessions from Benin and China using UPGMA (unweighted pair group method with arithmetic mean) based on 12 SSR markers.

An UPGMA (unweighted pair group method with arithmetic mean)‐based clustering analysis showed the similarity among the 19 accessions (Figure 7). The accessions were divided into three groups. The first group comprised local accessions SI01, SI08, SI11, SI02, SI12, SI04, SI13, SI06, and SI10. The second group is composed of Chinese accessions Y02, Y03, Y04, Y05, and Y06 and the third group included local and Chinese accessions SI03, SI05, SI09, SI07, and Y01.

#### 3.2.4 Relationship Between Agromorphological and Molecular Traits

The Mantel test showed that there was no significant correlation between the agromorphological and molecular data (*r* = 0.186, *p* > 0.05).

## 4 Discussion

This study investigated the molecular and agromorphological differentiation of 19 sesame accessions, including 13 Benin core collection with six exotic varieties, using 14 agromorphological descriptors and 12 SSR molecular markers. As suggested by Wu et al. (2014), the genetic diversity assessment, including landraces and exotic collections, is essential for effective parental line selection and the formulation of breeding strategies. The Chinese accessions used in this study are particularly significant because China is a leading sesame producer, with high‐yielding varieties averaging 1223 kg ha^−1^ in 2020 (Myint et al. 2020). In addition, this study focusing on genetic diversity assessment is of a great importance for the sesame value chain in Benin. As mentioned by Salem and Sallam (2016), understanding genetic diversity is a key to improve the economic value of crops. Consequently, this study offers insights into both Beninese and Chinese sesame collections, contributing to the selection of best parental accessions, with promising traits observed in the exotic and local accessions. Variation was observed in environmental conditions across locations (rainfall, relative humidity, and soil characteristics). Table S3 showed that there is a variation for the studied traits across environments, which could be linked to differences in environmental conditions.

### 4.1 Agromorphological Characterization

The assessment of agromorphological diversity provides essential agronomic prebreeding data. The ANOVA showed that there are significant differences among the accessions used in this study for almost all the traits studied except the NL. As mentioned by Sharma et al. (2020), this suggests a variability to be exploited for a breeding program. Dossa et al. (2016) studied the genetic relatedness among 96 sesame accessions collected from 22 countries and found that the genetic diversity observed in African accessions was lower than that in Asian accessions. The result obtained in our study is opposite to this finding where the Chinese accessions showed low genetic diversity for the studied traits. This was confirmed by the clustering analysis, which showed the difference between the Group 1 including Chinese accessions and the other clusters.

The PCA showed that the first component explained 52.36% of the total variation, and the traits, such as DE, D50F, CD, PH, NB, and SY, highly contributed to the variation among accessions. As Boampong et al. (2018) suggested, attention should be given to PH, D50F, and NB when aiming for SY improvement. The hierarchical clustering showed that the accessions were clustered into three groups. The first group involved the accessions of China with one local accession of Benin. This result is the opposite of Pandey et al. (2015) who suggested that the accession of the same origin should be gathered in the same cluster. In this case, geographical origin did not influence the clustering pattern. The accession SI09 was the accession in Benin core collection that had fewest branches, which could explain why it was included in the first group of accessions. All the accessions from China have SY values under 500 kg ha^−1^ and are nonbranched. This confirms the high correlation between the SY and the NB.

The average days for the early flowering date was 36.46 DAS. Similar values were obtained with Animasaun et al. (2022) and Sabag, Morota, and Peleg (2021) in two different countries, including Nigeria and Israel, respectively. These accessions could be selected as parents in case of development of early flowering accessions. As the trend is toward developing early flowering and high seed‐yielding cultivars, the Spearman correlation could help to identify the correlation between early flowering and agronomic traits. Considering the D50F and SY, the correlation test indicated that there is a positive and moderate correlation between the two traits. The same results were observed by Patel et al. (2023) and Brima et al. (2021). This suggests that the selection of early flowering accessions could be considered as selection of accessions with moderate values for SY. The average value obtained for SY ranged from 296.66 to 832.65 kg ha^−1^. The average yield obtained in Benin and reported by studies of Ajavon, Bello, and Adégbola (2015) was 449.91 kg ha^−1^, whereas the Department of Agricultural Statistics of the Ministry of Agriculture, Livestock, and Fisheries of Benin reported an average yield of 532 kg ha^‐1^ for 2019–2020 cropping season (DSA 2022).

In their work on the agromorphological characterization of cassava, Agre et al. (2015) emphasized the need for complementary studies, such as multienvironment trials across diverse agroecological zones and molecular characterization. We advocate for the assessment of stability in various environments, across years before selecting the best accessions, ensuring that the chosen accessions are both high performing and stable. Our research indicated that, with the exception of the local accession SI09, the accessions from Benin exhibit branching, whereas the Chinese accessions do not. This difference may account for the higher yields observed in the Benin accessions compared to those from China.

In summary, this study highlights the significance of Chinese accessions due to their early flowering characteristics, whereas the Benin accessions demonstrated the highest yield. It is recommended to explore the crossing of Chinese accessions with the high‐yielding Benin accessions to develop sesame cultivars that are both early flowering and high yielding for adoption in Benin cropping systems.

### 4.2 Molecular Characterization

Many genetic diversity studies carried out in sesame using SSR markers revealed low, moderate, and high diversity among the used germplasm. The total NA obtained in our study is 62 and ranged from 2 to 15 alleles per locus with an average value of 5.16 alleles. This NA was higher than what was obtained by Ramprasad et al. (2017) who used 41 sesame genotypes with 20 markers, but lower than what was previously obtained by Adu‐Gyamfi, Prempeh, and Zakaria (2019) using 25 sesame accessions with 21 markers. A maximum number of 134 alleles were obtained by Bhattacharjee et al. (2020) in genetic diversity assessment using 30 SSR markers and 18 accessions. The weak NA obtained in our study compared to the one of Bhattacharjee et al. (2020) despite the approximatively same number of genotypes used could be due to the weak number of markers (12) used in our study. A higher number of markers could better reveal the allele richness of our collection. Also, our collection has shown some diversity to be exploited due to the high NA obtained compared to the ones of Ramprasad et al. (2017).

In addition, the MAF, GD, and PIC values were moderate confirming the moderate genetic diversity in our collection. Less genetic diversity, mainly moderate and low, was also obtained by Ramprasad et al. (2017) and Harfi et al. (2021) who used, respectively, Indian and Moroccan accessions with 75 and 24 SSR markers. However, Ramprasad et al. (2017) suggested that the moderate level of diversity revealed by SSR markers in his study is only indicative and not conclusive due to the small number of loci analyzed. As suggested by Fu (2015), the increasing of sample and wider coverage of the genome would be of great importance in genetic diversity analysis. Considering the polymorphism information content, with the values of the markers obtained in our study (0.17–0.83), we find that these markers can be utilized in molecular diversity assessment in sesame, and as suggested by Chemutai et al. (2016), we can conclude based on the average value obtained in our study (0.55) that the markers used are reasonably informative. Only the marker M12 (ZMM4893) was slightly informative, whereas the remaining markers were reasonably or highly informative. The average NA obtained in our study is relatively close (4.90) to what was obtained by Dossa et al. (2016) using West African accessions, which did not include any Benin accessions. Park et al. (2014) having observed a high diversity in sesame core collection concluded that outcrossing could explain the observed richness of genetic diversity. The average GD value obtained in this study is 0.59. This value was lower than what was observed by Adu‐Gyamfi, Prempeh, and Zakaria (2019, GD = 0.91).

The genetic diversity in our study is higher than that of Harfi et al. (2021) who find low genetic diversity in sesame accessions from Morocco. We need to explore avenues for outcrossing to enhance the genetic diversity within the Benin core collection. This could involve introducing new germplasm from diverse origins for crossing or inducing mutations in existing germplasm.

The AMOVA showed that the variation was higher within population than that among population. The same results were obtained by Dossa et al. (2016) who used two different populations from Africa and Asia. The results could be explained by the fact that Chinese accessions have similarities with Benin collection, which showed the 8% of variation obtained in the AMOVA.

### 4.3 Relationship Between Agromorphological and Molecular Analyses

Our study showed that molecular and agromorphological clustering yielded different results. The result obtained for both molecular and agromorphological clustering showed significant variation among used accessions. The dendrogram obtained for molecular analysis showed three different groups. The first group includes only local accessions; the second group, only Chinese accessions; and the third, local accessions SI03, SI07, SI05, and SI09 with one Chinese accession (Y01). We can either conclude that the accessions from two origins, Benin and China, are close when considering the markers used or that this particular accession (Y01) is genetically close to Benin accessions when considering the markers used.

The same observation was made by Houmanat et al. (2023) in assessing the genetic diversity of rapeseed from Morocco and from various other origins. Their results showed that accessions from different origins belong to the same cluster. They concluded that Moroccan accessions are probably genetically close to the accessions with which they share the same cluster. The grouping of accession SI09 in the agromorphological dendrogram, with the Chinese accessions could be due to early flowering trait and lower number of branches observed in this accession, similar to Chinese accessions.

However, the Mantel test revealed a positive but not significant correlation between agromorphological and molecular data. The same result was obtained by Parsaeian, Mirlohi, and Saeidi (2011) in sesame genetic diversity assessment, which suggests that both agromorphological and molecular data are independent of each other. The non association of both agromorphological and molecular diversity could be due to many reasons. The agromorphological variation was derived from a small fraction of the genome and was influenced by environmental factors, whereas molecular diversity may not necessarily be linked to gene expression. In addition, the polymorphism could result from the coverage of both coding and noncoding genomic regions (Khan et al. 2009).

## 5 Conclusion

The genetic diversity study conducted in sesame collection of Benin and exotic accessions from China revealed diversity for both agromorphological and molecular characterization. The classification of agromorphological characters in three different groups shows the possibilities of selection among those groups. Traits such as DE, D50F, CD, PH, NB, and SY were the most discriminant among accessions for agromorphological characterization. The accessions such as SI08, SI12, SI13, SI11, SI06, SI04, SI05, and SI10 were revealed as the most promising accessions to be selected for the breeding program for yield‐related traits. A multienvironment trial test across years needs to be performed to confirm these accessions. A molecular analysis revealed similarities between Beninese and Chinese accessions. In addition, our study revealed that there is a weak correction between the agromorphological and molecular data. We suggest multiyear trials to have mega‐data in sesame in Benin to decipher genotype‐by‐environment interaction effect.

## Conflicts of Interest

The authors declare no conflicts of interest.

## Supporting information


Data S1.

## Data Availability

The data that supports the findings of this study are available in the Supporting Information of this article.
