# Supplementary material for: Molecular Diversity and Agronomic Performance of Sesame (Sesamum indicum) Cultivars in Benin: Local Cultivars and Lines Introduced From China
Source: Plant Environ Interact. 2024 Dec 24;5(6):e70024. doi: 10.1002/pei3.70024 (PMC11668925; doi:10.1002/pei3.70024)
Supplement: Supplementary file 1 — Data S1. [file PEI3-5-e70024-s001.docx]

**Supplementary tables**

**Table S1 :** Total variance explained

|  | **Eigen value** | **Variance (%)** | **cumulative.variance (%)** |
| --- | --- | --- | --- |
| **Axis 1** | 7.33 | 52.36 | 52.36 |
| **Axis 2** | 3.24 | 23.16 | 75.52 |
| **Axis 3** | 1.34 | 9.59 | 85.11 |
| **Axis 4** | 0.91 | 6.49 | 91.61 |
| **Axis 5** | 0.45 | 3.19 | 94.80 |
| **Axis 6** | 0.28 | 2.02 | 96.82 |
| **Axis 7** | 0.18 | 1.27 | 98.09 |
| **Axis 8** | 0.11 | 0.77 | 98.86 |
| **Axis 9** | 0.07 | 0.51 | 99.36 |
| **Axis 10** | 0.04 | 0.30 | 99.66 |
| **Axis 11** | 0.02 | 0.17 | 99.84 |
| **Axis 12** | 0.02 | 0.12 | 99.96 |
| **Axis 13** | 0.01 | 0.04 | 100.00 |
| **Axis 14** | 0.00 | 0.00 | 100.00 |

| **Table S2.** Principal component analysis of the agromorphological traits of the sesame accessions. | | | | | |
| --- | --- | --- | --- | --- | --- |
| **Traits** | **PC1** | **PC2** | **PC3** | **PC4** | **PC5** |
| DE | 0.31 | 0.26 | 0.47 | -0.76 | -0.11 |
| DFF | 0.94 | -0.12 | 0 | 0.17 | 0.01 |
| D50F | 0.95 | -0.25 | 0.05 | -0.11 | 0.07 |
| DCI | 0.95 | -0.27 | 0.01 | -0.05 | 0.03 |
| PH | 0.85 | 0.39 | -0.07 | 0.14 | 0.02 |
| NB | 0.96 | -0.24 | 0.03 | -0.05 | 0.09 |
| DC | 0.78 | 0.4 | -0.05 | 0.34 | -0.19 |
| NC | 0.83 | 0.41 | 0.03 | 0.06 | -0.13 |
| CL | -0.55 | 0.58 | -0.47 | -0.12 | 0.25 |
| CW | -0.8 | 0.36 | 0.35 | 0.2 | -0.2 |
| NL | -0.36 | -0.38 | 0.78 | 0.29 | 0.09 |
| NSC | 0.07 | 0.8 | 0.35 | 0.08 | 0.46 |
| TSW | -0.33 | 0.87 | -0.03 | -0.03 | -0.22 |
| SY | 0.68 | 0.69 | 0.17 | 0.03 | -0.02 |

**DE:** day to emergence. **DFF:** Day to first flowering. **D50F:** Day to 50% percent flowering. **DCI:** Day to capsule initiation. **PH:** Plant Height. **NB:** Number of branches**. NC:** Number of capsules. **CD:** Collar diameter. **CL:** Capsule length. **CW:** Capsule width. **NSP:** Number of capsules per pod. **NL:** Number of lodges. **TSW:** Thousand seed weight. **SY:** Seed Yield. **PC:** Principal Component

| **Table S3.** Description of the agromorphological traits used to characterise local and introduced sesame accessions in Benin | | | |  |
| --- | --- | --- | --- | --- |
| **Trait** | **Code** | **Description of the trait** | **Units** | |
| Days to 50% emergence | DE | Number of days from sowing to which 50% of the population in each plot emerged | Days after sowing | |
| Days to 50% flowering | D50F | Number of days from emergence to which 50% of the population in each plot become flowered. | Days after sowing | |
| Days to first flowering | DFF | Number of days from sowing to first flower appearance | Days after sowing | |
| Days to capsule initiation | DCI | Number of days from sowing to first capsule appearance | Days after sowing | |
| Collar diameter | CD | Recorded on ten central plants at the harvest | cm | |
| Plant height | PH | Length of plant from above ground to the terminal apex of the leaf | m | |
| Number of branches | NB | Total number of branches on the principal stem from the bottom to upper part of the plant | Count data | |
| Capsules plant ^-1^ | NC | Mean number of capsules from ten randomly selected plants in each plot | Count data | |
| Number of seed per pod | NSP | Mean number of seeds from ten randomly selected capsules from ten different plants in each plot | Count data | |
| Number of lodges per capsule | NL | Number of lodges per capsule bearing the seeds recorded on nine pods per plant of the ten central plants | Count data | |
| Capsule Length | CL | Mean value of the length of the nine capsules per plant. of the ten central plants | m | |
| Capsule width | CW | Mean value of the width recorded on nine capsules per plant on the ten central plants | cm | |
| 1000 Seed weight | TSW | Weight of 100 random dried seeds obtained and converted to 1000-seed weight by multiplying by 10. | g | |
| Seed yield hectare^-1^ | SY | Plot yield converted to hectare yield through the method of Nadeem et al.. (2015)  $SY=\frac{Seed weight per plot (kg)}{Plot size (m^{2})}*10000$ | kg. ha^-1^ | |

| **Table S4.** Coefficient of variation of studied variables per environment and across accessions | | | | | | | | | | | | | | |
| --- | --- | --- | --- | --- | --- | --- | --- | --- | --- | --- | --- | --- | --- | --- |
| **Environment** | **DE** | **DFF** | **D50F** | **DCI** | **PH** | **NB** | **CD** | **NC** | **CL** | **CW** | **NSP** | **NL** | **TSW** | **SY** |
| **Abomey-Calavi** | 33.31* | 33.31*** | 31.13*** | 30.43*** | 53.60*** | 54.52*** | 30.23*** | 58.81*** | 28.26** | 47.79*** | 18.01*** | 18 | 45.66*** | 49.03*** |
| **Sékou** | 34.88*** | 48.47*** | 32.82*** | 38.82*** | 51.07*** | 37.75*** | 53.79** | 35.79** | 46.79*** | 33.43*** | 52.74*** | 18 | 57.18*** | 50.03*** |
| **Tchaourou** | 21.69 | 48.47*** | 30.17*** | 55.32*** | 26.02.07*** | 47.75*** | 53.24*** | 15.89 | 19.9* | 33.17* | 26.81* | 18 | 52.16*** | 17.52* |
| **Djougou** | 36.47*** | 49.23*** | 30.50*** | 38.61*** | 42.70*** | 36.85*** | 43.61*** | 36.93** | 45.24*** | 45.24*** | 36.48*** | 18 | 33.42*** | 51.47** |
| **Boukoumbe** | 19.35 | 37.46*** | 35.53*** | 53.15*** | 50.15*** | 30.35*** | 51.84*** | 36.58*** | 46.32*** | 43.64*** | 28.50*** | 18 | 46.78*** | 33.60*** |
| **Matéri** | 22.96* | 47.23*** | 38.99*** | 51.84*** | 37.55*** | 30.99*** | 43.78** | 26.91 | 18.74** | 27.57*** | 22.23* | 18 | 47.26** | 35.49** |
| **Kerou** | 31.75* | 38.43*** | 55.84*** | 37.84*** | 18.38** | 39.86*** | 40.75*** | 31.87* | 38.84** | 50.48*** | 15.52** | 18 | 37.56*** | 34.25** |
| **Kandi** | 20.13* | 54.97*** |  | 31.57*** | 25.67*** | 56.79*** | 51.64** | 43.17** | 28.51** | 39.98*** | 20.20** | 18 | 53.41*** | 34.72** |
| **DE:** days to emergence. **DFF:** Day to first flowering. **D50F:** Days to 50% percent flowering. **DCI:** Days to capsule initiation. **PH:** Plant Height. **NB:** Number of branches**. NC:** Number of capsules per plant. **CD:** Collar diameter. **CL:** Capsule length. **CW:** Capsule width. **NSC:** Number of Seeds per capsule. **NL:** Number of lodges. **TSW:** Thousand seed weight. **SY:** Seed Yield.  *****: p<0.001; **: p<0.01; *: p<0.05** | | | | | | | | | | | | | | |

| **Table S5.** Mean. coefficient of variation of agromorphological variables across accessions and environments | | | | | | | | | | | | | | |
| --- | --- | --- | --- | --- | --- | --- | --- | --- | --- | --- | --- | --- | --- | --- |
|  | **DE** | **DFF** | **D50F** | **DCI** | **PH** | **NB** | **CD** | **NC** | **CL** | **CW** | **NSP** | **NL** | **TSW** | **SY** |
| **SI01** | 5.87a | 43.5a | 58.81ab | 53.62a | 91.12abc | 8.93ab | 13.63a | 55.85ab | 23.5e | 6.7de | 50.34d | 4a | 2.11e | 462.39abc |
| **SI02** | 6.31a | 33.9abc | 45.71cd | 39.45cdefg | 90.8abc | 4.46cd | 13.32a | 65.80a | 27.49abcde | 7.35bcde | 58.87abcd | 4a | 2.65bc | 749.89ab |
| **SI03** | 5.43b | 43.81a | 61a | 51.33ab | 95.96ab | 10.18a | 12.36a | 48.49ab | 23.62e | 6.59e | 54.75bcd | 4a | 2.17e | 555.54abc |
| **SI04** | 5.78a | 33.87abc | 45.06cde | 40.45cdef | 98.32ab | 4.53cd | 13.69a | 52.93ab | 29.35ab | 7.68abcd | 59.53abcd | 4a | 2.87abc | 658.16a |
| **SI05** | 5.84b | 33abc | 45.81cd | 39.7cdefg | 100.6a | 3.96cde | 14.51a | 60.37ab | 27.27abcde | 7.60abcde | 62.21abc | 4a | 2.86abc | 759.72ab |
| **SI06** | 5.25a | 33.71abc | 44.62cde | 41.04cd | 98ab | 3.81 | 13.04a | 50.02ab | 27.22abcde | 7.61abcde | 63.28ab | 4a | 2.88abc | 621.14bc |
| **SI07** | 5.81b | 42.18ab | 56.9ab | 50.33ab | 97.05ab | 8.9ab | 12.87a | 63.2ab | 24.16de | 6.73cde | 55.25bcd | 4a | 2.12e | 628.12ab |
| **SI08** | 5.78b | 38.81abc | 51.46bc | 45.91bc | 89.23abc | 7.09b | 16.54a | 66.60a | 25.23cde | 7.42bcde | 55.93abcd | 4.03a | 2.72bc | 740.47ab |
| **SI09** | 5.09a | 28.46bc | 38.84def | 34.58defg | 68.54abc | 2.18ef | 10.65a | 50.12ab | 30.76a | 7.69abcd | 58.28abcd | 4a | 2.6cd | 514.14abc |
| **SI10** | 6.28a | 34.31abc | 47.71c | 40.58cde | 95.01ab | 4.65cd | 13.15a | 58.51ab | 28.57abc | 7.82ab | 67a | 4.02a | 2.89abc | 832.65a |
| **SI11** | 6.53a | 31.4abc | 44.03cdef | 37.66defg | 78.7abc | 2.4def | 10.41a | 45.64ab | 26.61bcde | 7.94ab | 61.28abcd | 4a | 3.1a | 625.79ab |
| **SI12** | 5.09a | 34.62abc | 45.78cd | 40.79cd | 95.98ab | 3.75cde | 13.38a | 64.44a | 26.05bcde | 7.90ab | 56.71abcd | 4a | 2.92ab | 725.45ab |
| **SI13** | 5.21a | 45.51a | 45.8cd | 41.12cd | 99.74ab | 4.78c | 17.37a | 58.50ab | 26.35bcde | 7.60abcde | 61.4abcd | 4a | 2.75bc | 727.99ab |
| **Y01** | 5.46a | 26.84c | 37.56ef | 33.16g | 59.71c | 0.87f | 8.62a | 27.4b | 23.59e | 8.52a | 57.25abcd | 4.09a | 2.32de | 296.66b |
| **Y02** | 5.06a | 27.68c | 38.06def | 33.91defg | 77.3abc | 0.59f | 11.34a | 37.34ab | 29.7ab | 7.81ab | 56.71abcd | 4a | 2.64bc | 381.79ab |
| **Y03** | 5.53a | 27.62c | 38.46def | 33.5efg | 74.5abc | 0.87f | 10.35a | 37.61ab | 28.82abc | 7.86ab | 56.75abcd | 4a | 2.78bc | 464.40bc |
| **Y04** | 5.62a | 27.18c | 36.46f | 33.25fg | 74.65abc | 0.87f | 10.82a | 36.88ab | 28.09abcd | 8.19ab | 53.12bcd | 4a | 2.76bc | 392.51c |
| **Y05** | 5.34a | 27.03c | 37.28ef | 33.37efg | 65.59bc | 0.78f | 9.18a | 27.19b | 29.39ab | 7.55abcde | 55.78abcd | 4a | 2.66bc | 278.56bc |
| **Y06** | 5.34a | 27.4c | 38.28def | 33.37efg | 72.99abc | 0.67f | 9.63a | 32.98ab | 26.81abcde | 7.79abc | 51cd | 4a | 2.68bc | 332.91ab |
| **Means** | 5.62 | 33.71 | 45.14 | 39.85 | 85.47 | 3.91 | 12.37 | 49.47 | 26.98 | 7.6 | 57.66 | 4.05 | 2.66 | 565.7 |
| **CV** | 32.23 | 49.94 | 25.1 | 23.48 | 47.05 | 99.63 | 81.4 | 85.26 | 18.34 | 16.84 | 23.5 | 7.18 | 16.79 | 103.73 |

**DE:** days to emergence. **DFF:** Days to first flowering. **D50F:** Days to 50% percent flowering. **DCI:** Days to capsule initiation. **PH:** Plant Height. **NB:** Number of branches**.** **NC:** Number of capsules per plant. **CD:** Collar diameter. **CL:** Capsule length. **CW:** Capsule width. **NSC:** Number of Seeds per capsule. **NL:** Number of lodges. **TSW:** Thousand seed weight. **SY:** Seed Yield. **CV:** Coefficient of Variation

Data

| Agromorphological variables across accessions and environments | | | | | | | | | | | | | | |
| --- | --- | --- | --- | --- | --- | --- | --- | --- | --- | --- | --- | --- | --- | --- |
|  | **DE** | **DFF** | **D50F** | **DCI** | **PH** | **NB** | **CD** | **NC** | **CL** | **CW** | **NSP** | **NL** | **TSW** | **SY** |
| **SI01** | 5.87 | 43.5 | 58.81 | 53.62 | 91.12 | 8.93 | 13.63 | 55.85 | 23.5 | 6.7 | 50.34 | 4 | 2.11 | 462.39 |
| **SI02** | 6.31 | 33.9 | 45.71 | 39.45 | 90.8 | 4.46 | 13.32 | 65.80 | 27.49 | 7.35 | 58.87 | 4 | 2.65 | 749.89 |
| **SI03** | 5.43 | 43.81 | 61 | 51.33 | 95.96 | 10.18 | 12.36 | 48.49 | 23.62 | 6.59 | 54.75 | 4 | 2.17 | 555.54 |
| **SI04** | 5.78 | 33.87 | 45.06 | 40.45 | 98.32 | 4.53 | 13.69 | 52.93 | 29.35 | 7.68 | 59.53 | 4 | 2.87 | 658.16 |
| **SI05** | 5.84 | 33 | 45.81 | 39.7 | 100.6 | 3.96 | 14.51 | 60.37 | 27.27 | 7.60 | 62.21 | 4 | 2.86 | 759.72 |
| **SI06** | 5.25 | 33.71 | 44.62 | 41.04 | 98 | 3.81 | 13.04 | 50.02 | 27.22 | 7.61 | 63.28 | 4 | 2.88 | 621.14 |
| **SI07** | 5.81 | 42.18 | 56.9 | 50.33 | 97.05 | 8.9 | 12.87 | 63.2 | 24.16 | 6.73 | 55.25 | 4 | 2.12 | 628.12 |
| **SI08** | 5.78 | 38.81 | 51.46 | 45.91 | 89.23 | 7.09 | 16.54 | 66.60 | 25.23 | 7.42 | 55.93 | 4.03 | 2.72 | 740.47 |
| **SI09** | 5.09 | 28.46 | 38.84 | 34.58 | 68.54 | 2.18 | 10.65 | 50.12 | 30.76 | 7.69 | 58.28 | 4 | 2.6 | 514.14 |
| **SI10** | 6.28 | 34.31 | 47.71 | 40.58 | 95.01 | 4.65 | 13.15 | 58.51 | 28.57 | 7.82 | 67 | 4.02 | 2.89 | 832.65 |
| **SI11** | 6.53 | 31.4 | 44.03 | 37.66 | 78.7 | 2.4 | 10.41 | 45.64 | 26.61 | 7.94 | 61.28 | 4 | 3.1a | 625.79 |
| **SI12** | 5.09 | 34.62 | 45.78 | 40.79 | 95.98 | 3.75 | 13.38 | 64.44 | 26.05 | 7.90 | 56.71 | 4 | 2.92 | 725.45 |
| **SI13** | 5.21 | 45.51 | 45.8 | 41.12 | 99.74 | 4.78 | 17.37 | 58.50 | 26.35 | 7.60 | 61.4 | 4 | 2.75 | 727.99 |
| **Y01** | 5.46 | 26.84 | 37.56 | 33.16 | 59.71 | 0.87 | 8.62 | 27.4b | 23.59 | 8.52 | 57.25 | 4.09 | 2.32 | 296.66 |
| **Y02** | 5.06 | 27.68 | 38.06 | 33.91 | 77.3 | 0.59 | 11.34 | 37.34 | 29.7 | 7.81 | 56.71 | 4 | 2.64 | 381.79 |
| **Y03** | 5.53 | 27.62 | 38.46 | 33.5 | 74.5 | 0.87 | 10.35 | 37.61 | 28.82 | 7.86 | 56.75 | 4 | 2.78 | 464.40 |
| **Y04** | 5.62 | 27.18 | 36.46 | 33.25 | 74.65 | 0.87 | 10.82 | 36.88 | 28.09 | 8.19 | 53.12 | 4 | 2.76 | 392.51 |
| **Y05** | 5.34 | 27.03 | 37.28 | 33.37 | 65.59 | 0.78 | 9.18 | 27.19 | 29.39 | 7.55 | 55.78 | 4 | 2.66 | 278.56 |
| **Y06** | 5.34 | 27.4 | 38.28 | 33.37 | 72.99 | 0.67 | 9.63 | 32.98 | 26.81 | 7.79 | 51 | 4 | 2.68 | 332.91 |
